# Development of a unified system for assessing health related quality of life across the cancer care continuum: the EUonQoL Delphi study to identify priorities for quality of life domains

**DOI:** 10.1186/s41687-025-00907-z

**Published:** 2025-06-19

**Authors:** Maike G. Sweegers, Esther de Jongh, Christopher Bedding, Emma Nicklin, Daniela Doege, Sara Alfieri, Laura Gangeri, Bianca Scacciati, Augusto Caraceni, Cinzia Brunelli, Anne Bredart, Leslye Rojas-Concha, Helle Pappot, Giovanni Apolone, Nanne Bos, Gennaro Ciliberto, Norbert Couespel, Montse Ferrer, Stein Kaasa, Claudio Lombardo, Ricardo Pietrobon, Gabriella Pravettoni, Aude Sirven, Hugo Vachon, Mogens Groenvold, Maria Alice Franzoi, Galina Velikova, Alexandra Gilbert, Lonneke V. van de Poll-Franse

**Affiliations:** 1 Division of Psychosocial Research and Epidemiology, The Netherlands Cancer Institute, Amsterdam, The Netherlands; 2 Leeds Institute of Medical Research at St James’s, University of Leeds, Leeds, UK; 3 Unit of Cancer Survivorship, Division of Clinical Epidemiology and Aging Research, German Cancer Research Center (DKFZ), Heidelberg, Germany; 4 Clinical Psychology Unit, Fondazione IRCCS Istituto Nazionale dei Tumori, Milan, Italy; 5 Università degli studi di Milano Dipartimento di Scienze Cliniche e di Comunità, Dipartimento di Eccellenza, 2023-2027, Milan, Italy; 6 Palliative Care, Pain Therapy and Rehabilitation Unit, Fondazione IRCCS Istituto Nazionale Tumori-Milano, Milan, Italy; 7 Psycho-Oncology Unit, Institut Curie, Paris, France; 8 Psychology Institute, Psychopathology and Health Process Laboratory UR4057, ED 261, Paris City University, Paris, France; 9 Palliative Care Research Unit, Department of Geriatrics and Palliative Medicine GP, Copenhagen University Hospital - Bispebjerg and Frederiksberg, University of Copenhagen, Copenhagen, Denmark; 10 Department of Oncology, Copenhagen University Hospital – Rigshospitalet, Institute of Clinical Medicine, University of Copenhagen, Copenhagen, Denmark; 11 Scientific Directorate, Fondazione IRCCS Istituto Nazionale Dei Tumori (INT), Milan, Italy; 12 Netherlands Institute for Health Services Research (Nivel), Utrecht, The Netherlands; 13 IRCCS National Cancer Center “Regina Elena”, on Behalf of DIGICORE, Rome, Italy; 14 European Cancer Organisation (ECO), Brussels, Belgium; 15 Health Services Research Group, Hospital del Mar Research Institute, Barcelona, Spain; 16 CIBER en Epidemiología y Salud Pública, CIBERESP, Madrid, Spain; 17 Department of Medicine and Life Sciences, Universitat Pompeu Fabra, Barcelona, Spain; 18 Oslo Universitetssykehus HF (OUS), Oslo, Norway; 19 Organisation of European Cancer Institutes (OECI), Brussels, Belgium; 20 SporeData OÜ, Tallinn, Estonia; 21 Istituto Europeo Di Oncologia IRCCS, Milan, Italy; 22 Unicancer, Paris, France; 23 European Organisation for Research & Treatment of Cancer, Quality of Life Department, Brussels, Belgium; 24 Section of Health Services Research, Department of Public Health, University of Copenhagen, Copenhagen, Denmark; 25 Cancer Survivorship Group, Inserm U981, Villejuif, France; 26 Department of Medical and Clinical Psychology, Tilburg University, Tilburg, The Netherlands

**Keywords:** Quality of life, Cancer, Delphi study, Patient-reported outcome measures, Patient centred research

## Abstract

**Introduction:**

Cancer and cancer treatment have a major impact on health related quality of life (HRQoL). To improve the assessment of HRQoL in patients with cancer and evaluate the impact of policy interventions, the European Oncology Quality of Life (EUonQoL) project aims at developing a digital, patient centred system to assess HRQoL based on evaluations and preferences of cancer patients and survivors: the EUonQoL-kit.

**Method:**

Patients across the cancer care continuum, healthcare professionals and researchers from six European countries (Denmark, France, Germany, Italy, The Netherlands and United Kingdom) were asked to rate the importance of 44 pre-selected HRQoL subdomains over a maximum of three Delphi survey rounds. We evaluated the importance of HRQoL subdomains for three target populations: patients undergoing active treatment, cancer survivors and patients receiving palliative care. The results were discussed during a consensus meeting.

**Results:**

96 patients and 59 healthcare professionals participated in the Delphi study. After three rounds, consensus was reached for 20 subdomains: *ability to work*,* communication with healthcare professionals*,* diarrhoea*,* fatigue*,* fear of recurrence*,* global health status*,* impact of treatment side effects*,* impact on children/family*,* insomnia*,* instrumental activities of daily living*,* maintaining independence*,* mobility*,* nausea*,* overall quality of life*,* pain*,* partner relationship*,* social activity limitations*,* social isolation*,* symptom awareness* and *uncertain prognosis.* The subdomains pain and fear of recurrence were rated as important for all three target populations.

**Conclusion:**

Subdomains that were considered important for the assessment of HRQoL in patients with cancer can be summarised into: physical symptoms, mobility & activity, future outlook, social roles & activities, family & relationships, social isolation, self-efficacy, overall HRQoL, and healthcare experience. The importance of the subdomains differed for patients in different phases of the cancer care continuum. These findings were used for the creation of the first version of the EUonQoL-Kit, as a base for its further development.

**Supplementary Information:**

The online version contains supplementary material available at 10.1186/s41687-025-00907-z.

## Introduction

Patients with cancer experience many health issues related to their disease or treatment, which may negatively affect their health related quality of life (HRQoL) [1, 2]. The World Health Organization defines QoL as *‘an individual’s perception of their position in life in the context of the culture and value systems in which they live and in relation to their goals*,* expectations*,* standards and concerns’* [3]. Multiple instruments have been designed to measure HRQoL, some of which are specifically designed to evaluate HRQoL in patients with cancer [4, 5]. 

Regular assessment of HRQoL provides information on the physical, psychological, and social changes due to treatment or disease progression in patients with cancer. This information may help policymakers, authorities, and healthcare providers to identify inequalities and monitor policy interventions to meet patients’ needs. Most HRQoL assessment tools are generic, or either disease- or treatment-specific and instruments have seldom been used to identify HRQoL inequalities and patient subgroups who may benefit from specific cancer care policy interventions on an international level. Due to advances in cancer treatment, more patients receive intensive and long treatments, resulting in improved survival [6]. Therefore, a revision of traditional questionnaires is necessary, personalizing them to patients in distinct phases of the disease. Furthermore, a broad stakeholder involvement, including patients’ perspectives, is necessary to improve QoL assessments’ relevance, uptake and impact [7]. 

The current manuscript presents a subproject of the ‘Quality of Life in Oncology: measuring what matters for cancer patients and survivors in Europe (EUonQoL)’ project [8, 9]. This project aims to develop, pilot and validate the EUonQoL-kit; a patient driven, unified system to assess HRQoL in patients with cancer and survivors. Here, we present the results of a Delphi study, in which we collected information on European patients’ priorities for HRQoL subdomains and aimed at identifying potentially missing subdomains across the cancer care continuum. Based on the findings of this Delphi study, systematic reviews [10–12], patient interviews and usability testing [13, 14], the EUonQoL-kit will be developed in 25 European and six associated countries’ languages for psychometric testing. The final EUonQoL-kit will be used in periodic surveys and policymaking in the field of oncology across Europe.

## Method

The Delphi study was conducted in six countries as part of the EUonQoL-kit development project (Denmark, France, Germany, Italy, The Netherlands and United Kingdom; UK). A Delphi study is a research method used to achieve consensus by collecting expert opinions [15]. The Delphi study was conducted in order to (further) concentrate on the most important HRQoL domains and address potential shortcomings of existing instruments. The same study procedures were applied in all countries to allow for collective analysis of the results from each centre. The Delphi study was conducted in compliance with the Declaration of Helsinki, the Data Protection Act (1998), Local Research Ethics Committee approval and other regulatory requirements as appropriate.

### Identification of relevant HRQoL subdomains

To ensure the validity of the new HRQoL measure from the beginning of the development process, an intensive review of the available literature was conducted. Prior to the Delphi study, two systematic reviews and a scoping review on Computerized Adaptive Testing (CAT) measurement were conducted to provide a comprehensive overview of the available patient-reported outcome measurement systems in oncology [10, 11]. These reviews provided input for the development of the EUonQoL conceptual framework, consisting of the domains *social health*, *physical health*, *psychological wellbeing* and *overall health*. Findings from these reviews and input from co-researchers (i.e. persons that had been diagnosed with any kind of cancer, or their caregivers, who collaborated with the researchers in the EUonQoL project) were used to inform the list of HRQoL subdomains to be used in the Delphi study [16]. For subdomains, example questions were identified from the EORTC Item Library and Patient Reported Outcomes Measurement Information System (PROMIS), and conceptual models [17–19]. A complete list of the subdomains and example questions included in the Delphi study is presented in Table 1. In parallel with the Delphi study, 75 individual patient interviews were conducted to identify patient preferences and priorities for HRQoL domains and subdomains, the results of which are presented elsewhere [13]. 

**Table 1:** Subdomains and example questions presented in the Delphi study

Dimension	Subdomain	Example question	Source
Physical symptoms	Pain/pain interference	Have you had pain?	EORTC – Q12
	Fatigue/Energy	Have you lacked energy?	EORTC – Q159
	Insomnia	Have you had trouble sleeping?	EORTC – Q14
	Appetite loss	Have you lacked appetite?	EORTC – Q16
	Nausea	Have you felt nauseated?	EORTC – Q17
	Constipation	Have you been constipated?	EORTC – Q19
	Diarrhoea	Have you had diarrhoea?	EORTC – Q20
	Dyspnoea	Were you short of breath?	EORTC – Q11
	Sensory neuropathy	Have you had tingling or numbness in your hands or feet?	EORTC – Q462
	Symptom awareness	How much has your disease been a burden to you?	EORTC – Q46
	Impact of treatment side-effects	To what extent have you been troubled with side-effects from your treatment?	EORTC – Q168
Mobility & activity	Mobility	Do you need to stay in bed or a chair during the day?	EORTC – Q8
	Physical exercise	Do you have any trouble taking a long walk?	EORTC – Q5
	Activities Daily Living	Do you need help with eating, dressing, washing yourself or using the toilet?	EORTC – Q9
	Instrumental Activities Daily Living	Do you have any trouble doing strenuous activities, like carrying a heavy shopping bag or a suitcase?	EORTC – Q4
Sex life	Sexual problems	Has the treatment affected your sexual activity?	EORTC – Q88
	Sexual pleasure	Was sexual activity enjoyable for you?	EORTC – Q84
Body image	Body image	Have you been dissatisfied with your physical appearance?	EORTC – Q981
Anxiety & Worry	Anxiety	Did you worry?	EORTC – Q25
	Depression	Did you feel depressed?	EORTC – Q27
Psychological distress & stress	Distress	Have you felt stressed?	EORTC – Q164
Future outlook	Fear of recurrence	Have you worried about recurrence of your disease?	EORTC – Q364
	Uncertain prognosis	Have you been worried about your health in the future?	EORTC – Q41
	Future Life plans	Have you had to limit your life plans or goals?	EORTC – Q988
Memory & concentration	Cognitive problems	Have you had difficulty remembering things?	EORTC – Q28
Positive impact	Positive affect	Has the experience of cancer helped you to distinguish between important and unimportant things in life?	EORTC – Q1007
	Positive life outlook	Have you had a positive outlook on life in the last week?	EORTC – Q44
Spirituality	Spirituality	I have felt at peace with myself	EORTC – Q596
Meaning & purpose	Meaning & purpose	Do you feel that your life has more purpose?	EORTC – Q1005
Social roles & activities	Ability to Work	Were you limited in doing either your work or other daily activities?	EORTC – Q7
	Leisure activities -Hobbies	Were you limited in pursuing your hobbies or other leisure time activities?	EORTC – Q10
	Leisure travel	Have you been limited in your ability to travel?	EORTC – Q529
	Social activity limitations	Has your physical condition or medical treatment interfered with your social activities?	EORTC – Q30
Family & relationships	Impact on children/family	Has your physical condition or medical treatment interfered with your family life?	EORTC – Q29
	Fertility: Ability to have children	Have you been concerned about your ability to have children?	EORTC – Q155
	Partner relations	Is your relationship with your partner stronger?	EORTC – Q1004
Social isolation & connectivity	Social isolation	Have you felt isolated from those close to you (e.g. family, friends)?	EORTC – Q719
	Social support	I have felt able to share thoughts about life with people who are close to me.	EORTC – Q601
Self-efficacy	Self-efficacy	Have you lacked self-confidence?	EORTC – Q517
	Maintaining independence	Have you worried that you are a burden to other people?	EORTC – Q294
Financial aspects	Financial difficulties	Has your physical condition or medical treatment caused you financial difficulties?	EORTC – Q31
	Insurance	Have you had problems with obtaining insurance, loans, and/or a mortgage?	EORTC – Q1011
Overall quality of life	Overall quality of life	How would you rate your overall quality of life during the past week?	EORTC – Q33
Overall health perspective	Global health status	How would you rate your overall health during the past week?	EORTC – Q32
**New items included in Round 2**			
Healthcare experience	Communication with healthcare professionals	Have you been satisfied with your communication with your professional(s)?	EORTC – Q429
Changes in weigth	Changes in weight	Have you been concerned about changes in your weight?	EORTC – Q375
Lifestyle changes	Lifestyle changes	Have you made positive lifestyle changes (e.g. more exercise, healthy food, cutting down smoking?	EORTC – Q1012

### Identification of relevant stakeholders

The aim of the EUonQoL-kit development is to assess HRQoL in all patients with a cancer diagnosis and cancer survivors. In order to identify the most important HRQoL subdomains across the cancer care continuum, we defined three target populations:

- *Active treatment*: Patients who are undergoing or recently completed curative treatment for early-stage cancers or undergoing non-curative treatment for advanced cancers.
- *Survivors*: Patients who are disease-free without evidence of active cancer, and at least one year off active treatment (with the exception of long-term adjuvant hormonotherapy).
- *Palliative care*: Patients with advanced cancer who meet at least one of the following criteria:
  - *Prognosis < 12 months with a Karnofsky Performance Status (KPS) < 70.*
  - *Referred to a specialist palliative care team for symptom control.*
  - *Receiving non-curative treatment purely for symptom control.*

These definitions might not be mutually exclusive or exhaustive of the whole population of people living with or beyond cancer. However, we aimed at distinguishing three target populations with relative precision to validate the EUonQoL-kit.

Relevant stakeholders for the Delphi study included cancer patients and survivors in these different target populations with different types of cancer, as well as healthcare professionals (i.e., medical specialists, primary care physicians, nurses and nursing specialists, clinical exercise professionals and researchers). Although the EUonQoL-kit will be used to assess HRQoL in cancer patients and survivors, healthcare professionals might prioritize HRQoL subdomains differently from a clinical perspective. We considered the inclusion of a wide range of perspectives to be important for the development of the toolkit. Stakeholders were eligible if they were: aged 18+, able and willing to give informed consent, able to read and understand the local language of the countries in which the Delphi was conducted, and did not exhibit overt psychopathology or serious cognitive dysfunction that would prevent them from participating in the study. Patient participation did not affect the treatment or care of the patient.

Within each country, local EUonQoL collaborators identified relevant stakeholders by: (1) directly contacting medical specialists, nurses or other healthcare professionals involved in cancer care, (2) asking medical specialists to identify eligible patients, (3) identifying patients from cancer registries, and/or (4) asking patient representatives involved in cancer research to identify potentially eligible participants. We aimed to include 25–35 patients per patient target population and an additional 30 healthcare professionals.

### Registration

Potential participants received an invitation letter and those who agreed to participate received a link to an online, country specific registration page. Here, participants provided consent to participate in the Delphi survey by ticking a box ‘I agree to participate in and receive email notifications regarding this study’. In the invitation letter, participants were informed that by ticking this box at registration, they provide consent to participate in the study. In countries where the local ethical committees did not approve digital consent, additional written informed consent was collected.

As some participants might be unaware of their treatment phase according to our definitions, we instructed them to register to the target population they felt was applicable to them. They were presented with the following definitions: (A) *someone undergoing or recently completed treatment for cancer*, (B) *as someone who is at least one year off treatment and without evidence of active cancer* or (C) *as someone with advanced cancer*,* who may be referred to a specialist palliative care team or receiving palliative treatment to control symptoms*. Healthcare professionals were instructed to register for group A, B or C depending on their main field of expertise, and received instructions to indicate the importance of HRQoL subdomains for patients in that specific target population. Although healthcare professionals often work with several target populations, requiring them to reflect on all pre-selected QoL items for the three target populations was considered too burdensome.

### Delphi process

After online registration, all participants received an online invitation for the first Delphi round. We used the online software program DelphiManager (COMET, 2016) to conduct the Delphi study. This software is designed to facilitate the management of Delphi surveys and the pseudonymisation of the data. The research team did not have access to the key files and was unable to identify participants’ individual survey responses. As such, the Delphi survey was conducted in accordance with the EU law regarding the general data protection regulation (GDPR). The Delphi survey was made available in six languages (Danish, Dutch, English, German, French and Italian).

The participants were invited to the first Delphi round via email in May 2023. In this first round, background information was collected using a brief questionnaire. Healthcare professionals provided details about their current job roles, and patients were asked to provide basic sociodemographic and clinical data (i.e., marital status, employment status, education level, cancer type and treatment).

All participants were asked to rate the proposed HRQoL subdomains on a 9-point Likert scale, based on the Grading of Recommendations, Assessment, Development and Evaluations (GRADE) method [20], with scorings 1–3 representing *limited importance*, 4–6 *important but not critical* and 7–9 meaning *critically important* for inclusion in the EUonQoL kit. Each subdomain was accompanied by an example question.

In the first round, participants were given the opportunity to suggest new subdomains they considered important and were not yet included in the list. Suggested subdomains that were mentioned more than once were discussed with the research team after completion of the first round and, if deemed relevant, the subdomain was included in the second round of the Delphi survey.

In the second round, for each subdomain that did not reach consensus in the first round, the participants were provided with a summary of the results from the first round for each target population, as well as their own response. This allowed participants to reflect and adjust their ratings. Participants were also asked to rate the newly included subdomains. The same scoring system (1–9 scale) was used to score the subdomains in the second round.

In case consensus on all outcomes was reached after the second round, no subsequent survey round would have been organized. However, as no consensus was reached for certain subdomains, a third round was organized. The study ended after the pre-determined maximum of three rounds, irrespective of whether consensus had been reached for all subdomains. Participants were asked to complete each round within 2 weeks and participants received automatic reminders through DelphiManager if they did not complete the survey round after one week.

### Analysis

After each round, we evaluated whether consensus was reached for any of the subdomains within each target population (i.e., active treatment, survivors, or palliative care). In doing so, we combined the responses from patients and healthcare professionals for each group. Subdomains that were rated as *critically important* (scoring 7 to 9) by at least 75% of the participants within one group and as of limited importance (1 to 3 points) by 15% or less of the participants within one group were considered to have reached consensus on importance. With these criteria, the majority agrees that the subdomain is essential and only a small minority considers the subdomain to be of limited importance [5]. We applied a conservative approach during these consensus discussions, by evaluating responses in all groups and incorporating findings from the systematic reviews and interviews. Subdomains rated as critically important by approximately 70% of the participants within one group were discussed with the research team. When a decision was made that consensus was reached in one group, the subdomain was not included for evaluation in the next round.

Findings from the Delphi study, the systematic reviews and patient interviews were used as a basis for the discussion on the relevance of different HRQoL subdomains. This discussion took place during a consensus meeting including co-researchers, the EUonQoL multi-professional stakeholder board and researchers from the EUonQoL consortium.

## Results

In total, 155 cancer patients, survivors, and healthcare professionals participated in the Delphi study. Fifty-five participants allocated themselves to the active treatment group, 74 to the survivor group, and 26 to the palliative care group (Table 2). Due to the stringent timelines of this EU project and the limited time available to complete the Delphi study, the UK were unable to participate in the first round, however did so in subsequent rounds after obtaining ethical approval. Although some subdomains were rated as critically important by more than 75% of the participants in the active treatment group and the palliative care group from the remaining countries, we decided not to exclude any subdomain after the first round. This allowed participants from the UK to provide their rating on all subdomains in the second round. Recruitment of patients for the palliative care group was challenging and we were unable to recruit the target number of patients for this specific group. We recruited more participants for the other target populations to compensate for drop out. Most participants were female and aged 40–59 years (Table 3).

**Table 2:** Number of participants in the Delphi study

Target population	Round 1			Round 2			Round 3		
	Patients	HCPs	Total	Patients	HCPs	Total	Patients	HCPs	Total
Active treatment (A)	26 (54%)	22 (46%)	**48**	19 (58%)	14 (42%)	**33**	12 (41%)	17 (59%)	**29**
Survivors (B)	47 (70%)	20 (30%)	**67**	31 (80%)	8 (20%)	**39**	35 (73%)	13 (27%)	**48**
Palliative care (C)	12 (55%)	10 (45%)	**22**	8 (50%)	8 (50%)	**16**	9 (56%)	7 (44%)	**16**

HCPs = healthcare professionals

**Table 3:** Participants’ characteristics

Characteristic	*N*	Active treatment A	Survivors B	Palliative Care C
**All participants**				
**Age**	155	*N* = 55	*N* = 74	*N* = 26
18–39 years		11 (20%)	19 (26%)	1 (3.8%)
40–59 years		21 (38%)	28 (38%)	15 (58%)
60 + years		23 (42%)	27 (36%)	10 (38%)
**Sex**	155	*N* = 55	*N* = 74	*N* = 26
Female		37 (67%)	45 (61%)	18 (69%)
Male		18 (33%)	29 (39%)	8 (31%)
**Country**	155	*N* = 55	*N* = 74	*N* = 26
Denmark		6 (11%)	11 (15%)	8 (31%)
France		2 (3.6%)	21 (28%)	1 (3.8%)
Germany		13 (24%)	19 (26%)	4 (15%)
Italy		17 (31%)	5 (6.8%)	3 (12%)
Netherlands		10 (18%)	11 (15%)	6 (23%)
United Kingdom		7 (13%)	7 (9.5%)	4 (15%)
**Health care professionals only**				
**Job**	59	*N* = 25	*N* = 22	*N* = 12
Medical specialist		4 (16%)	8 (36%)	7 (58%)
Mental health specialist		3 (12%)	4 (18%)	1 (8.3%)
Nurse		4 (16%)	2 (9.1%)	1 (8.3%)
Nursing specialist		3 (12%)	1 (4.5%)	1 (8.3%)
Physical activity specialist		0 (0%)	1 (4.5%)	0 (0%)
Primary care physician		1 (4.0%)	0 (0%)	0 (0%)
Researcher		3 (12%)	6 (27%)	2 (17%)
Other		7 (28%)	0 (0%)	0 (0%)
**Patients only**				
**Marital status**	96	*N* = 30	*N* = 52	*N* = 14
Divorced		2 (6.7%)	6 (12%)	0 (0%)
Married/living together		21 (70%)	30 (58%)	11 (79%)
Partner living apart		0 (0%)	5 (9.6%)	2 (14%)
Single		5 (17%)	11 (21%)	0 (0%)
Widowed		2 (6.7%)	0 (0%)	0 (0%)
Missing		0 (0%)	0 (0%)	1 (7.1%)
**Education level**	96	*N* = 30	*N* = 52	*N* = 14
College or University		20 (67%)	43 (83%)	8 (57%)
High school		10 (33%)	5 (9.6%)	3 (21%)
None/ primary school only		0 (0%)	4 (7.7%)	2 (14%)
Missing		0 (0%)	0 (0%)	1 (7.1%)
**Employment status**	96	*N* = 30	*N* = 52	*N* = 14
Disabled		1 (3.3%)	1 (1.9%)	0 (0%)
Full time		10 (33%)	22 (42%)	2 (14%)
Homemaker		1 (3.3%)	3 (5.8%)	0 (0%)
Part-time		1 (3.3%)	2 (3.8%)	1 (7.1%)
Retired		12 (40%)	18 (35%)	4 (29%)
Student		0 (0%)	2 (3.8%)	0 (0%)
Unemployed		3 (10%)	2 (3.8%)	0 (0%)
Other		2 (6.7%)	2 (3.8%)	6 (43%)
Missing		0 (0%)	0 (0%)	1 (7.1%)
**Cancer type**	96	*N* = 30	*N* = 52	*N* = 14
Bladder cancer		1 (3.3%)	0 (0%)	2 (14%)
Breast cancer		8 (27%)	15 (29%)	3 (21%)
Colorectal cancer		1 (3.3%)	6 (12%)	2 (14%)
Gynaecological cancer		1 (3.3%)	4 (7.7%)	1 (7.1%)
Head and neck cancer		4 (13%)	7 (13%)	1 (7.1%)
Lung cancer		3 (10%)	4 (7.7%)	1 (7.1%)
Lymphoma		1 (3.3%)	3 (5.8%)	0 (0%)
Melanoma		2 (6.7%)	0 (0%)	0 (0%)
Prostate cancer		2 (6.7%)	4 (7.7%)	2 (14%)
Testicular cancer		0 (0%)	3 (5.8%)	0 (0%)
Other		7 (23%)	6 (12%)	1 (7.1%)
Missing		0 (0%)	0 (0%)	1 (7.1%)

### Results from the first Delphi round

Data from 137 participants were used for the analysis of the first Delphi round. Forty-eight participants were allocated to the active treatment group, 67 to the survivor group and 22 to the palliative care group. In the first Delphi round, 75% or more of the participants in the active treatment group considered *overall QoL* to be critically important. More than 75% of the participants in the palliative care group considered *fatigue*, *impact of treatment side effects*, *insomnia*, *overall QoL* and *pain* to be critically important. None of the subdomains in the survivor group were considered important by more than 75%. During the first round, several additional subdomains were suggested by participants. Three newly suggested subdomains were included in the second Delphi round: *communication with healthcare professionals*, *changes in weight* and *lifestyle changes.*

### Results from the second Delphi round

Eighty-eight participants responded to the second round of the Delphi study (33 in the active treatment group, 39 in the survivor group and 16 in the palliative care group). The following subdomains were considered critically important by at least 75% of participants within one group and were not included in the third Delphi round: *fatigue*, *fear of recurrence*, *impact of treatment side effects*, *impact on children/family*, *insomnia*, *mobility*, *overall QoL*, *pain*, *partner relationship*, *social isolation*, *uncertain prognosis*. Multiple subdomains were considered important by more than 70% of the participants in at least one group. After discussion, the following subdomains were considered to have reached consensus and were also not included in the third round: *ability to work*, *communication with healthcare professionals*, *global health status* and *maintaining independence*.

### Results from the third Delphi round

Ninety-three participants responded to the third round (29 in the active treatment group, 48 in the survivor group and 16 in the palliative care group). In the third round, only the subdomain *social activity limitations* was considered important by more than 75% in one of the groups (active treatment group). Another four subdomains were considered important by approximately 70% in one of the groups and were considered to have reached consensus after discussion: *diarrhoea*, *instrumental activities of daily living*, *nausea*, and *symptom awareness*. Some subdomains were considered important by approximately 70% of the participants in one of the groups but were not considered to have reached consensus after discussion. These subdomains were: *activities of daily living*, *anxiety*, *cognitive problems*, *dyspnoea*, and *physical exercise*. The percentages of participants rating the subdomains of critical importance are presented in Table 4.

**Table 4:** Percentages of participants considering an item of critical importance (rating 7–9) in the final round of the Delphi study

	Consensus	Active treatment A (%)	Survivors B (%)	Palliative Care C (%)
**Physical symptoms**				
Pain/pain interference	ROUND 2	**75**	**76**	**85**
Fatigue/Energy	ROUND 2	69	59	**92**
Insomnia	ROUND 2	59	65	**92**
Appetite loss	No	29	34	53
Nausea	ROUND 3	61	51	67
Constipation	No	50	34	60
Diarrhoea	ROUND 3	54	38	*73*
Dyspnoea	No	*71*	43	60
Sensory neuropathy	No	57	40	27
Symptom awareness	ROUND 3	*71*	40	*73*
Impact of treatment side-effects	ROUND 2	*72*	68	**85**
**Mobility & activity**				
Mobility	ROUND 2	62	59	**85**
Physical exercise	No	*71*	57	*73*
Activities Daily Living	No	*71*	43	67
Instrumental Activities Daily Living	ROUND 3	*71*	49	67
**Sex life**				
Sexual problems (physical)	No	43	38	33
Sexual pleasure	No	21	30	27
**Body image**				
Body image	No	39	55	33
**Anxiety & worry**				
Anxiety	No	*71*	64	56
Depression	No	64	60	56
**Psychological distress & stress**				
Distress	No	61	53	50
**Future outlook**				
Fear of progression/recurrence	ROUND 2	*72*	*74*	**85**
Uncertain prognosis	ROUND 2	**78**	**76**	62
Future life plans	No	64	55	62
**Memory & concentration**				
Cognitive problems	No	*71*	68	62
**Positive impact**				
Positive affect	No	39	43	40
Positive life outlook	No	41	40	40
**Spirituality**				
Spirituality	No	22	15	7
**Meaning & purpose**				
Meaning and purpose	No	52	36	40
**Social roles & activities**				
Ability to Work	ROUND 2	61	*73*	54
Leisure activities -Hobbies	No	62	52	67
Leisure travel	No	24	27	20
Social activity limitations	ROUND 3	**79**	58	*73*
**Family & relationships**				
Impact on children/family	ROUND 2	**75**	59	**85**
Fertility: Ability to have children	No	25	28	20
Partner relations	ROUND 2	69	65	**92**
**Social isolation & connectivity**				
Social isolation	ROUND 2	**75**	65	**77**
Social support	No	64	62	53
**Self-efficacy**				
Self-efficacy	No	68	53	67
Maintaining independence	ROUND 2	69	*73*	62
**Financial aspects**				
Financial difficulties	No	68	55	47
Insurance	No	21	34	33
**Overall quality of life**				
Global health status	ROUND 2	69	*73*	60
Overall quality of life	ROUND 2	**78**	68	**87**
**New items**				
Communication with healthcare professionals	ROUND 2	53	*73*	69
Changes in weight	No	36	51	53
Lifestyle changes	No	68	55	50

Ratings of critical importance by 70–74% of participants are presented in italics; Ratings of critical importance by 75–100% of participants are presented in bold

### Consensus meeting

After the final round, the findings from the Delphi study were discussed with the members of the EUonQoL consortium in October 2023. Here we discussed the percentages of participants who rated each subdomain as critically important, as presented in supplementary Table 1. We aimed at developing version 1 of the EUonQoL-kit, consisting of three distinct questionnaires; one for each target population. More details about the process of triangulation of the results from all EUonQoL subprojects into version 1 of the toolkit are presented elsewhere [13]. In short, the subdomains that were considered of critical importance were grouped into the following HRQoL dimensions: physical functioning, role functioning, emotional functioning, social functioning, physical symptoms, financial difficulties, patient-reported experience measures and overall health. Appropriate questions for these dimensions (identified from the EORTC Item Library, PROMIS, the Danish Palliative Care Questionnaire, the Chronic Cancer Experiences Questionnaire and newly formulated items) were included in version 1 of the EUonQoL-kit. The subdomains *fertility*,* sexual pleasure* and *spirituality* were discussed at length during the consensus meeting. The relatively older patient population included in the Delphi study was considered as one of the potential reasons for *fertility* being rated of low importance (80% of participants were 40 years or older). For this reason, we still recommend the inclusion of an item related to *fertility* as part of the social health domain. Based on findings from the patient interviews, we decided to refer to intimacy and closeness rather than *sexual pleasure* to make the item more generalizable for all patients. No clear decision could be made regarding *spirituality* during the consensus meeting, due to heterogeneous findings. For this reason, the subdomain was included in version 1 of the EUonQoL-kit in order to collect more information on its importance. Version 1 of the EUonQoL-kit was tested in all three target populations in a usability study with debriefing interviews. The results were incorporated in version 2 of the EUonQoL-kit and we integrated CAT methodology where feasible to allow further customization. Version 2 is being piloted in 25 EU and six EU affiliated countries in 2024 [14]. An overview of the EUonQoL-kit development process is presented in Supplementary Fig. 1.

## Discussion

We performed a Delphi study to determine the importance of subdomains for the assessment of HRQoL in patients with cancer and survivors. One hundred and fifty-five patients and healthcare professionals from six European countries rated the importance of pre-selected HRQoL subdomains in three survey rounds. Consensus was reached on 20 of the 47 subdomains in the Delphi study. Through the evaluation of the importance of HRQoL subdomains, we conclude that relevant HRQoL dimensions are: physical symptoms, mobility & activity, future outlook, social roles & activities, family & relationships, social isolation, self-efficacy, overall QoL, and healthcare professional communication.

### QoL assessment in different target populations

Previously, HRQoL questionnaires have been developed with high reliability and validity, including questionnaires specifically for patients with cancer or survivors [4, 21, 22]. Rather than duplicating these efforts, we aimed to complement them by identifying important HRQoL subdomains in three different target populations: patients undergoing treatment, survivors and those receiving palliative care. This allows a more personalized assessment of patients’ HRQoL in distinct phases of the disease. Consensus was reached on several subdomains within each group; however, the subdomains that deemed critically important varied between groups. *Pain* and *fear of recurrence* were the only subdomains that were considered important for all groups. Six subdomains were considered important by two groups and 12 subdomains were considered important by only one group. For example, the item *uncertain prognosis* reached consensus in the active treatment and survivor group, whereas *symptom awareness* reached consensus in the active treatment and palliative care group. Such differences may reflect different needs and priorities for patients in different phases of their disease, which should be considered in HRQoL assessment. Recently, the EORTC QoL group has developed a specific questionnaire for disease free cancer survivors [23]. Similar to our findings, cancer survivors one year or longer after treatment rated acute symptoms as a result of cancer and cancer treatment as less important [24]. Differences in the importance of subdomains across target populations support the need for tailored QoL assessments adjusted to the different phases in the cancer care continuum.

Providing patients with the most appropriate questionnaire introduces a new complexity. There might be a discrepancy between a patients’ self-identification of their target population and the clinician’s assessment of a patient’s situation. A clinician might rate patients receiving long-term adjuvant hormonotherapy as belonging to the survivor group, while patients might identify themselves as someone undergoing active treatment for their cancer. Similarly, patients may find it difficult to understand or accept their current situation. One patient explained his situation as being ‘a combination of group A and B’; “*I have been under treatment for thyroid cancer since 2005 and I recently completed radiotherapy treatment for metastases in the L2 vertebra. I was also treated for prostate cancer in 2017*,* with good results*”. Besides the fact that these target populations are not mutually exclusive, the perception of the patient is arguably most important when assessing their HRQoL, as HRQoL is predominantly a subjective construct. Future studies in this field may consider relying solely on the patients’ perception.

### Additional items

In the first Delphi round, the participants suggested to include subdomains related to *communication with healthcare professionals*,* changes in weight* and *lifestyle changes*. Although patients with cancer or survivors may be prone to an unhealthy lifestyle and changes in weight [25], no consensus was reached for these two subdomains. On the contrary, the subdomain *communication with healthcare professionals* was considered critically important in the second round of the Delphi study. While this domain may not be traditionally associated with QoL, we did recommend the inclusion of an item related to healthcare communication in the EUonQoL-kit. Previously, quality of life was found to be associated with a patient’s satisfaction with care [26], and an external observer’s rating of the physician’s anxiety/nervousness was found to negatively affect patients’ global QoL [27]. However, no association was found between the physicians’ friendliness, dominance or socio-emotional behaviour and the patients’ QoL [27]. Healthcare professionals are expected to understand the patient’s verbal understanding, grasp their emotional needs, and communicate appropriately [28]. As the number of treatment options increases, so does the importance of the shared decision making approach. This approach requires effective communication in a highly complex situation, to ensure the co-creation of a mutually supported care plan [29]. For these reasons, we recommended to include an item related to a patients’ satisfaction with the communication with the healthcare professionals in the EUonQoL-kit.

### Strengths & limitations

Strengths of the current Delphi study include its international approach and the large number of participants included. Furthermore, we included a wide range of stakeholders to provide a variety of perspectives on important HRQoL subdomains for patients with cancer in different phases of the disease. There are several difficulties in attempting to identify differences in the importance of HRQoL across multiple target populations in a Delphi study in different countries. Although we were able to show the ratings of subdomains that did not reach consensus stratified by target population, participants saw the results for all three target populations. Knowledge of how subdomains were rated by participants in other target populations may have influenced participants’ responses. Furthermore, the DelphiManager software did not allow items to be excluded for a specific target population. Therefore, if consensus was reached in one of the groups, the subdomain was excluded for all groups in the next round. When discussing the importance of subdomains for each target population in the development of the EUonQoL-kit, the responses to the subdomains from all target populations in all rounds were considered, in addition to the findings from the systematic reviews and patient interviews. In doing so, the importance of all subdomains was considered equally for each target population, independent of whether consensus was reached. We recommend future studies using the DelphiManager software to develop independent projects for each target population. This allows the exclusion of subdomains only for the target population in which consensus was reached, and the presentation of the ratings from previous rounds for each target population separately. However, the benefit of setting up separate projects should be weighed against the added project management load and costs. Another limitation of the Delphi study was the result of the limited available time to conduct the study. Participants from the UK joined Round 2 of the Delphi study and automatically received feedback from Round 1, which could have biased their initial perception of the importance of HRQoL subdomains. However, because we used a conservative approach regarding decisions about consensus and were generous with subdomain inclusion, we expect the effect of the delayed inclusion of UK participants on our findings to be minimal. An advantage to the short time span in which the Delphi study was conducted was that it is unlikely that a patient switched between treatment phases, and thus target population during the data collection period. Finally, while we are aiming at developing a tool to be used across Europe, the lack of data from Eastern EU countries may limit the generalizability. The EUonQoL-kit is being piloted in 25 EU and six EU affiliated countries to identify potential cultural and linguistic differences that could impact its applicability.

## Conclusion

One hundred fifty-five stakeholders rated the importance of HRQoL subdomains in three Delphi rounds. Stakeholders were selected to represent target populations: patients undergoing active treatment, cancer survivors and patients receiving palliative care. Subdomains that were considered of critical importance belonged to the following HRQoL dimensions: social roles & activities, physical symptoms, physical function, family & relationships, independence, and health outlook, with differences across target populations. These findings were used to develop version 1 of the EUonQoL-kit, a patient-centred tool to assess HRQoL in patients with cancer and survivors.

## Electronic supplementary material

Below is the link to the electronic supplementary material.

Supplementary Material 1

Supplementary Material 2

## Data Availability

The authors declare that they have full control of all primary data and data will be made available on reasonable request.
