# Supplementary figures and images for: Development of a unified system for assessing health related quality of life across the cancer care continuum: the EUonQoL Delphi study to identify priorities for quality of life domains
Source: J Patient Rep Outcomes. 2025 Jun 19;9:70. doi: 10.1186/s41687-025-00907-z (PMC12179011; doi:10.1186/s41687-025-00907-z)

| 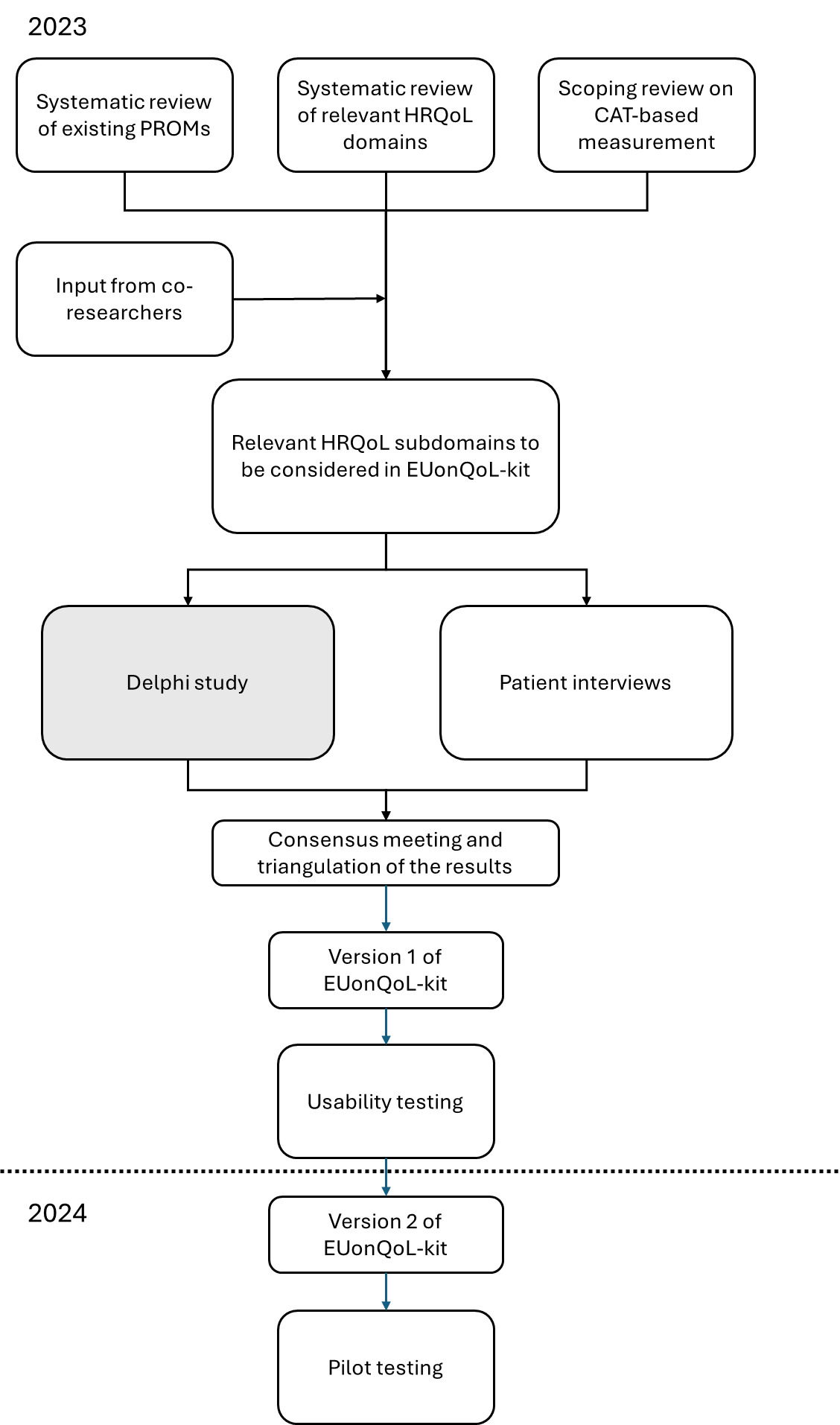 |
| --- |
| Supplementary figure 1. Study flow diagram of the EUonQoL project. |

Supplement: Supplementary file 1 — Supplementary Material 1 [file 41687_2025_907_MOESM1_ESM.docx]
