# Supplementary material for: Development of a unified system for assessing health related quality of life across the cancer care continuum: the EUonQoL Delphi study to identify priorities for quality of life domains
Source: J Patient Rep Outcomes. 2025 Jun 19;9:70. doi: 10.1186/s41687-025-00907-z (PMC12179011; doi:10.1186/s41687-025-00907-z)
Supplement: Supplementary file 2 — Supplementary Material 2 [file 41687_2025_907_MOESM2_ESM.docx]

| Supplementary table 1. Percentages of participants considering a subdomain of critical importance (rating 7–9) in all rounds of the Delphi study. | | | | | | | | | |
| --- | --- | --- | --- | --- | --- | --- | --- | --- | --- |
|  | ROUND 1 | | | ROUND 2 | | | ROUND 3 | | |
|  | A  N = 48 | B  N = 67 | C  N = 22 | A  N = 33 | B  N = 39 | C  N = 16 | A  N = 29 | B  N = 48 | C  N = 16 |
| Physical symptoms | | | | | | | | | |
| Pain/pain interference^2^ | 62 | 64 | 77 | 75 | 76 | 85 | n.a. | n.a. | n.a. |
| Fatigue/Energy^2^ | 64 | 57 | 86 | 69 | 59 | 92 | n.a. | n.a. | n.a. |
| Insomnia^2^ | 49 | 67 | 73 | 59 | 65 | 92 | n.a. | n.a. | n.a. |
| Appetite loss | 38 | 38 | 55 | 38 | 30 | 54 | 29 | 34 | 53 |
| Nausea | 53 | 44 | 73 | 59 | 46 | 69 | 61 | 51 | 67 |
| Constipation | 40 | 36 | 64 | 50 | 24 | 69 | 50 | 34 | 60 |
| Diarrhoea | 38 | 41 | 55 | 50 | 32 | 69 | 54 | 38 | 73 |
| Dyspnoea | 56 | 51 | 59 | 56 | 41 | 69 | 71 | 43 | 60 |
| Sensory neuropathy | 49 | 52 | 36 | 56 | 43 | 46 | 57 | 40 | 27 |
| Symptom awareness | 58 | 43 | 59 | 69 | 38 | 69 | 71 | 40 | 73 |
| Impact of treatment side-effects^2^ | 60 | 57 | 86 | 72 | 68 | 85 | n.a. | n.a. | n.a. |
| Mobility & activity | | | | | | | | | |
| Mobility^2^ | 62 | 56 | 73 | 62 | 59 | 85 | n.a. | n.a. | n.a. |
| Physical exercise | 56 | 57 | 59 | 59 | 57 | 69 | 71 | 57 | 73 |
| Activities Daily Living | 53 | 52 | 68 | 62 | 51 | 69 | 71 | 43 | 67 |
| Instrumental Activities Daily Living | 58 | 48 | 55 | 69 | 51 | 62 | 71 | 49 | 67 |
| Sex life | | | | | | | | | |
| Sexual problems (physical) | 44 | 48 | 32 | 47 | 43 | 31 | 43 | 38 | 33 |
| Sexual pleasure | 29 | 38 | 23 | 25 | 32 | 23 | 21 | 30 | 27 |
| Body image | | | | | | | | | |
| Body image | 44 | 52 | 32 | 47 | 51 | 31 | 39 | 55 | 33 |
| Anxiety & worry | | | | | | | | | |
| Anxiety | 62 | 57 | 64 | 72 | 63 | 64 | 71 | 64 | 56 |
| Depression | 51 | 63 | 55 | 62 | 66 | 62 | 64 | 60 | 56 |
| Psychological distress & stress | | | | | | | | | |
| Distress | 53 | 54 | 55 | 15 | 61 | 62 | 61 | 53 | 50 |
| Future outlook | | | | | | | | | |
| Fear of progression/recurrence^2^ | 64 | 65 | 45 | 72 | 74 | 85 | n.a. | n.a. | n.a. |
| Uncertain prognosis^2^ | 68 | 51 | 55 | 78 | 76 | 62 | n.a. | n.a. | n.a. |
| Future life plans | 64 | 54 | 55 | 69 | 59 | 62 | 64 | 55 | 62 |
| Memory & concentration | | | | | | | | | |
| Cognitive problems | 51 | 52 | 50 | 56 | 65 | 69 | 71 | 68 | 62 |
| Positive impact | | | | | | | | | |
| Positive affect | 49 | 43 | 41 | 47 | 51 | 54 | 39 | 43 | 40 |
| Positive life outlook | 55 | 42 | 36 | 53 | 38 | 46 | 41 | 40 | 40 |
| Spirituality | | | | | | | | | |
| Spirituality | 19 | 25 | 32 | 28 | 22 | 15 | 22 | 15 | 7 |
| Meaning & purpose | | | | | | | | | |
| Meaning and purpose | 32 | 45 | 41 | 44 | 46 | 54 | 52 | 36 | 40 |
| Social roles & activities | | | | | | | | | |
| Ability to Work^2^ | 50 | 63 | 59 | 61 | 73 | 54 | n.a. | n.a. | n.a. |
| Leisure activities -Hobbies | 54 | 46 | 41 | 58 | 46 | 54 | 62 | 52 | 67 |
| Leisure travel | 30 | 27 | 23 | 27 | 27 | 23 | 24 | 27 | 20 |
| Social activity limitations | 63 | 51 | 55 | 67 | 57 | 69 | 79 | 58 | 73 |
| Family & relationships | | | | | | | | | |
| Impact on children/family^2^ | 70 | 63 | 73 | 75 | 59 | 85 | n.a. | n.a. | n.a. |
| Fertility: Ability to have children | 37 | 37 | 27 | 22 | 27 | 15 | 25 | 28 | 20 |
| Partner relations^2^ | 61 | 59 | 59 | 69 | 65 | 92 | n.a. | n.a. | n.a. |
| Social isolation & connectivity | | | | | | | | | |
| Social isolation^2^ | 63 | 54 | 59 | 75 | 65 | 77 | n.a. | n.a. | n.a. |
| Social support | 59 | 57 | 73 | 62 | 57 | 62 | 64 | 62 | 53 |
| Self-efficacy | | | | | | | | | |
| Self-efficacy | 70 | 43 | 50 | 66 | 46 | 69 | 68 | 53 | 67 |
| Maintaining independence^2^ | 70 | 60 | 55 | 69 | 73 | 62 | n.a. | n.a. | n.a. |
| Financial aspects | | | | | | | | | |
| Financial difficulties | 59 | 60 | 41 | 69 | 68 | 46 | 68 | 55 | 47 |
| Insurance | 35 | 41 | 23 | 38 | 35 | 31 | 21 | 34 | 33 |
| Overall quality of life | | | | | | | | | |
| Overall quality of life^2^ | 76 | 58 | 77 | 78 | 68 | 87 | n.a. | n.a. | n.a. |
| Global health status^2^ | 70 | 65 | 68 | 69 | 73 | 60 | n.a. | n.a. | n.a. |
| New items | | | | | | | | | |
| Communication with healthcare professionals^1,2^ | n.a. | n.a. | n.a. | 53 | 73 | 69 | n.a. | n.a. | n.a. |
| Changes in weight^1^ | n.a. | n.a. | n.a. | 44 | 49 | 54 | 36 | 51 | 53 |
| Lifestyle changes^1^ | n.a. | n.a. | n.a. | 47 | 54 | 54 | 68 | 55 | 50 |

^1^no scoring available in round 1 because new items were added to the list in round 2.

^2^no scoring available in round 3 because consensus was reached in round 2.
